# Supplementary figures and images for: Complex Evolutionary and Genetic Patterns Characterize the Loss of Scleral Ossification in the Blind Cavefish Astyanax mexicanus
Source: PLoS One. 2015 Dec 9;10(12):e0142208. doi: 10.1371/journal.pone.0142208 (PMC4674125; doi:10.1371/journal.pone.0142208)

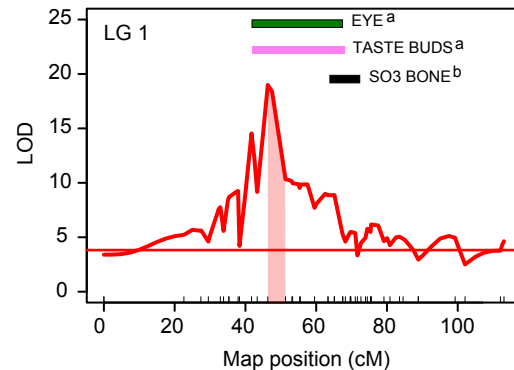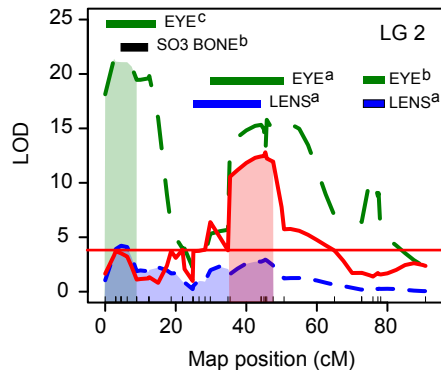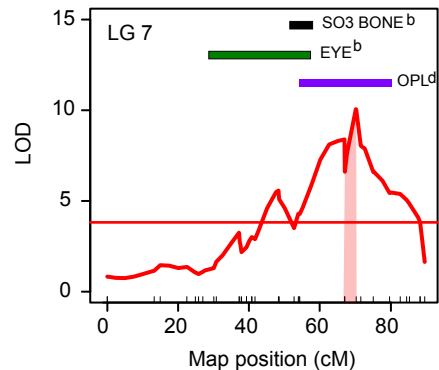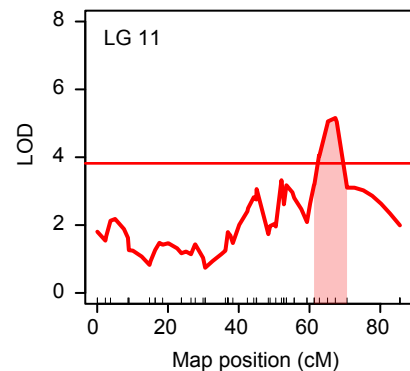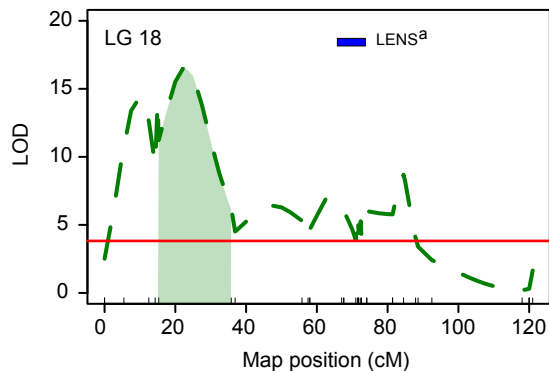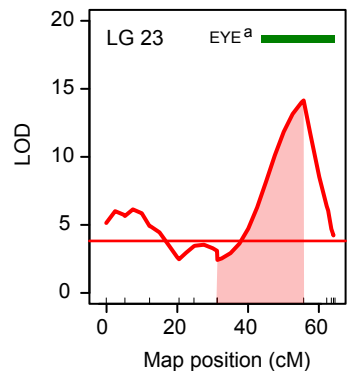

--- Pupil Area

--- Eye Diameter

— Eye Area

Supplement: S1 Fig — Detailed view of the eye or pupil size QTL on Astyanax linkage groups (LG) 1, 2, 7, 11, 18, and 23. Red line indicates P = 0.05; any value above that threshold is considered statistically significant. Shaded fields indicate the 95% Bayesian confidence interval for the location of each QTL. Colored boxes at the top of the chart indicate the position of eye, lens, retina, or bone QTL found in other studies. a = Protas et al. [44], b = Protas et al. [45], c = O'Quin et al. [29]. Two QTL found here–one for eye area on LG 11 and another for eye diameter on LG 18 –have not previously been described in other studies. (PDF) [file pone.0142208.s001.pdf]
