# Supplementary material for: Complex Evolutionary and Genetic Patterns Characterize the Loss of Scleral Ossification in the Blind Cavefish Astyanax mexicanus
Source: PLoS One. 2015 Dec 9;10(12):e0142208. doi: 10.1371/journal.pone.0142208 (PMC4674125; doi:10.1371/journal.pone.0142208)
Supplement: S3 Table — (DOCX) [file pone.0142208.s004.docx]

**S3 Table. Summary statistics for all significant QTL found in this study.**

| Phenotype | LG | QTL Peak (cM) | 95% BCI (cM) | LOD | P-value | PVE (%) | Dominance Deviation^a^ | Additive Effect^a^ | Dominance Effect^a^ |
| --- | --- | --- | --- | --- | --- | --- | --- | --- | --- |
| Ossified Sclera (Thres.) | 1 | 59.5 | 53.1 – 112.0 | 3.88 | 0.033 | 7.62 | 0.295 | 1.951 | -1.418 |
| Scleral Width / Diameter | 2 | 0.00 | 0.00 – 78.1 | 3.68 | 0.064 | 8.63 | 0.040 | 0.067 | 0.068 |
| Eye Diameter (mm) | 2 | 2.5 | 0.00 – 8.93 | 21.4 | <0.0001 | 42.1 | 0.090 | -0.097 | 0.116 |
|  | 18 | 22.5 | 15.3 – 35.8 | 16.6 | <0.0001 | 29.4 | 0.042 | -0.053 | 0.099 |
|  | 2:18 | -- | -- | 14.6 | <0.0001 | 24.6 | -- | -- | -- |
| Eye Area (mm^2^) | 1 | 46.3 | 46.3 – 51.3 | 19.0 | <0.0001 | 25.9 | 0.085 | -0.236 | 0.161 |
|  | 2 | 45.4 | 35.4 – 47.6 | 12.8 | <0.0001 | 15.3 | 0.040 | -0.090 | 0.122 |
|  | 7 | 70.0 | 66.8 – 70.1 | 10.1 | <0.0001 | 11.3 | 0.061 | -0.043 | -0.003 |
|  | 11 | 67.1 | 61.2 – 70.4 | 5.16 | <0.0001 | 5.23 | -0.017 | -0.110 | 0.021 |
|  | 23 | 55.6 | 31.3 – 55.6 | 14.1 | <0.0001 | 17.34 | 0.165 | -0.099 | 0.169 |
|  | 1:2 | -- | -- | 10.8 | <0.0001 | 12.36 | -- | -- | -- |
|  | 7:23 | -- | -- | 9.50 | <0.0001 | 10.55 | -- | -- | -- |
| Pupil/Lens Area (mm^2^) | 2 | 4.5 | 0.00 – 47.6 | 4.22 | <0.0001 | 14.16 | 0.174 | -0.161 | 0.221 |

^a^To make the effects of different QTL comparable for traits measured on different scales, we scaled the dominance deviation, additive effect, and dominance effect of each QTL based on the phenotypic mid-point between individuals with SFSF and CFCF genotypes at the peak QTL marker. These midpoints are: Ossification@LG1 = 0.654; Scleral Width@LG2 = 30.891; Eye Diameter@LG2 = 2.64 mm; Eye Diameter@LG18 = 2.72 mm; Eye Area@LG1 = 6.56 mm^2^; Eye Area@LG2 = 6.56 mm^2^; Eye Area@LG7 = 6.44 mm^2^; Eye Area@LG11 = 6.72 mm^2^; Eye Area@LG23 = 6.12 mm^2^; Pupil Area@LG2 = 1.71 mm^2^
